# Development of a microneedle patch for delivery of mRNA-lipid nanoparticles

**DOI:** 10.1007/s13346-025-01964-z

**Published:** 2025-09-02

**Authors:** Sophia H. Sakers, B. Pradeep K. Reddy, Gianna Fiduccia, Katherine E. Byrne, Ingrid Stén, Julie Kim, Afsane Radmand, James E. Dahlman, Mark R. Prausnitz

**Affiliations:** 1 Wallace H. Coulter Department of Biomedical Engineering, Georgia Institute of Technology and Emory University School of Medicine, Atlanta, GA USA; 2 School of Chemical and Biomolecular Engineering, Georgia Institute of Technology, Atlanta, GA USA; 3 KTH Royal Institute of Technology, Stockholm, Sweden

**Keywords:** Microneedle patch, mRNA, Lipid nanoparticle, Transdermal delivery

## Abstract

mRNA delivered by microneedle patch (MNP) can enable painless delivery, reduced need for healthcare expertise, and improved thermostability. In this study, we investigated formulation and manufacturing approaches for developing MNPs that deliver mRNA-loaded lipid nanoparticles (LNPs) encoding luciferase as a reporter protein during MNP fabrication and storage, including mRNA-LNP concentration, formulation, pH, excipients, and backing material. MNPs were assessed for mRNA-LNP size, encapsulation efficiency, and protein expression in vitro and in vivo. MNPs fabricated with mRNA-LNPs initially prepared at a higher concentration yielded superior expression compared to mRNA-LNP concentration by centrifugation or tangential flow filtration. Acidic pH during MNP manufacturing enabled greater expression in vitro. However, no such correlation was observed in vivo. Polyvinyl alcohol (PVA) best stabilized mRNA-LNPs during the MNP manufacturing process amongst the tested polymers. Incorporating sugars in MNPs did not further improve stability. Low temperature drying (5 °C) preserved mRNA functionality better compared to drying at 25 °C and 40 °C. Though there was significant activity loss initially (87% loss in 2 days at 40 °C), mRNA expression was stabilized for extended subsequent periods even at accelerated conditions (10% additional loss after 28 days at 40 °C). Our systematic approach identified key parameters for successful formulation and manufacturing approaches to incorporate mRNA-LNPs into MNPs, which could expand access to mRNA-based medical interventions.

**Supplementary Information:**

The online version contains supplementary material available at 10.1007/s13346-025-01964-z.

## Graphical Abstract

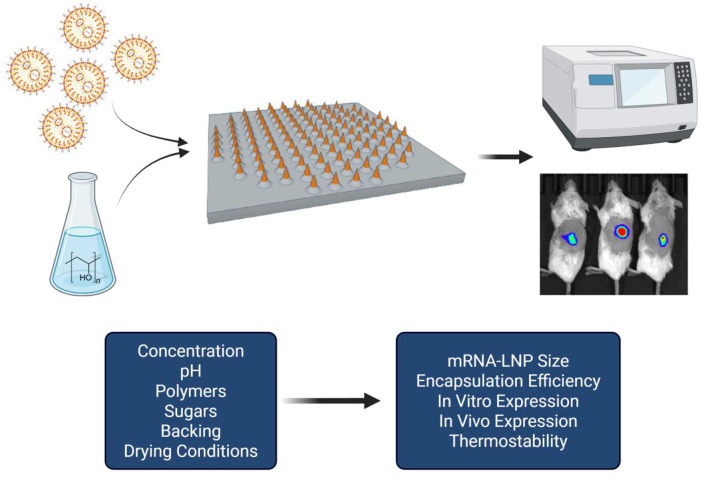

## Introduction

mRNA emerged as a powerful technology with uses in vaccination and genetic therapy. Compared to other types of vaccines and biologics, mRNA design and manufacturing requires fewer steps, enabling rapid and cost-effective production of novel mRNA vaccines [1–3] and therapeutics, particularly in cancer treatment [4–6]. Despite these advantages, therapies using naked mRNA are limited by low transfection efficiency, degradation, and non-durable immune responses [7, 8]. Several delivery vehicles have been developed for mRNA to address these limitations, including cationic polymers, cell-penetrating peptides, liposomes, lipopolyplexes, cationic LNPs, and ionizable LNPs [9, 10]. Amongst the various platforms, the ionizable LNPs have demonstrated superior transfection efficiency, endosomal escape, and mRNA stability [11, 12].

Ionizable lipid nanoparticles (LNPs) are nanoparticles containing an ionizable lipid, cholesterol, a phospholipid, and a PEGylated lipid [13, 14]. The crucial component, ionizable lipid, is cationic at acidic pH and binds to negatively charged mRNA [15, 16]. Several ionizable mRNA-LNP products have received USFDA-approval, including patisiran (Onpattro^®^) for the treatment of hereditary transthyretin-mediated amyloidosis and BNT162b2 (Comirnaty^®^) and mRNA-1273 (Spikevax^®^) vaccines against SARS-CoV-2 [17]. mRNA vaccines gained significant scientific interest in the wake of the COVID-19 pandemic.

Microneedle patches (MNPs) contain micron-scale needle arrays that can be used to deliver drugs or vaccines into the skin [18–21]. Dissolvable MNPs contain drugs/vaccines embedded in a matrix made of water-soluble polymers and other excipients that dissolve upon application to the skin and release their cargo [22]. MNP technology possesses distinct advantages, including minimally invasive delivery, painless delivery, elimination of sharps waste, and simplified administration with minimal training [23–25]. Finally, as a dried formulation, MNPs can increase the thermostability of drugs and vaccines [26, 27]. However, MNP development has challenges. For example, dissolvable MNPs have a limited capacity and are not well-suited for drugs that require a large dose (e.g., > 10 mg) [28]. In addition, the MNP manufacturing process involves air-drying, which could be detrimental to fragile biomolecules, like mRNA. The development of a MNP for delivering mRNA-LNPs would need to overcome these challenges.

MNP technology that could deliver mRNA vaccines would be particularly useful in a pandemic scenario, when there is a need for a low-cost, thermostable, and easy-to-administer delivery method [29]. Additionally, as more mRNA technologies are developed, advancement of delivery methods such as MNPs will enable faster introduction and a wider reach of these new vaccines and therapeutics. Since the structure of mRNA molecules is relatively consistent, basic research on MNPs for mRNA delivery should be broadly applicable to mRNA products. While mRNA can encode any protein, it may still be stabilized by similar formulations, unlike protein-based drugs that may require different formulations for each product. However, the stability profile may vary depending on the length of the mRNA and specific modifications made during synthesis [30].

There has been limited work on delivering mRNA via MNP. In 2018, Koh et al. delivered naked mRNA in a dissolvable MNP [31]. In 2022, Yu et al. incorporated transfection agents, including polyethyleneimine and liposomes, into a cryomicroneedle for mRNA delivery [32]. In 2023 and 2025, Jaklenec et al. delivered mRNA-LNPs via a dissolvable MNP [33, 34]. In 2024, Rajesh et al. delivered liquid mRNA-LNPs via a lattice MNP [35]. Despite these initial demonstrations of mRNA delivery by MNP, there remains a need to broadly focus on the factors that affect mRNA-LNP stability in a dissolvable MNP in terms of formulation, manufacturing, and resulting properties, such as mRNA-LNP size, mRNA encapsulation efficiency, and reporter protein expression in vitro and in vivo. This study aims to systematically investigate these attributes of a mRNA-LNP MNP, identifying factors that influence mRNA-LNP characteristics and mRNA expression to guide continued development of MNP products for mRNA-LNP delivery.

## Methods

### mRNA

Polyadenylic acid (PolyA; Cytiva, Marlborough, MA) was used as a substitute for mRNA in Fig. 2a and Supplementary Fig. 4. Firefly luciferase-encoding mRNA with N1’-methyl pseudouridine modification was obtained from ARNAV Biotech (Columbus, GA) for Figs. 2b, 3, 4, 6, 7, 8, 9, 10 and 11a-c, Supplementary Fig. 1, and Supplementary Fig. 8 and GenScript Biotech (Piscataway, NJ) for Figs. 5 and 11d-f. Nanoluciferase-encoding mRNA was obtained from the laboratory of Philip Santangelo (Georgia Tech) for Supplementary Fig. 2 and Supplementary Fig. 5–7.

### LNP fabrication

SM-102 ionizable lipid was obtained from BroadPharm (San Diego, CA). Cholesterol was obtained from Sigma-Aldrich (St. Louis, MO). 1,2-distearoyl-sn-glycero-3-phosphocholine (DSPC) and 1,2-dimyristoyl-rac-glycero-3-methoxypolyethylene glycol-2000 (DMG-PEG 2000) were obtained from Avanti Lipids (Alabaster, AL). LNPs were fabricated using a microfluidic chip as described previously [36]. Briefly, SM-102, cholesterol, DSPC, and DMG-PEG 2000 were dissolved in ethanol at a molar ratio 50:38.5:10:1.5, respectively, to make the ethanol phase. The aqueous phase consisted of PolyA/mRNA in 20 mM citrate buffer (100 mM, pH 3.0, Teknova, Hollister, CA diluted to 20 mM with RNase-free water). The SM-102:mRNA mass ratio was maintained at 15:1 or 20:1. The aqueous phase and ethanol phase were combined in a 3:1 flow rate ratio in a microfluidic chip, described previously [37]. For precise control of the flow rates, Pump 11 Elite syringe pumps (Harvard Apparatus, Holliston, MA) were used with FlowControl software (Harvard Apparatus).

### pH modification

For experiments measuring effect of pH, phosphate buffers (20 mM) were prepared by dissolving potassium phosphate monobasic (Fisher Scientific, Waltham, MA) to RNase-free water. Buffer pH was adjusted to 3.0, 4.5, 6.0, or 7.4 using o-phosphoric acid (Fisher Scientific) or 0.2 M NaOH (Fisher Scientific). mRNA-LNPs were diluted 40-fold in appropriate buffer and subsequently concentrated using centrifugal filtration at 4 °C (see below). The pH of the buffers was measured using a pH probe (Mettler Toledo, Columbus, OH) and verified in the concentrated samples using pH strips (VWR Chemicals, Radnor, PA).

### LNP concentration

LNPs were concentrated using centrifugal filtration, tangential flow filtration (TFF), and reverse dialysis. For centrifugal filtration, LNPs were diluted 40-fold with phosphate-buffered saline (PBS, Corning, Glendale, AZ) or tris(hydroxymethyl)aminomethane buffer (MilliporeSigma, Burlington, MA) and added to a Vivaspin 100 kDa MWCO filter (Cytiva, Marlborough, MA). They were spun at 1000 rcf and 4 °C until they reached the intended volume (e.g., ~ 120 min to reduce volume from ~ 40 mL to ~ 0.2 mL).

For TFF, MICROKROS 20CM 300KDa MPES TFF tubes from Repligen (Waltham, MA) were used. LNPs were diluted 3-fold with PBS or RNase-free water and drawn into a 5 mL syringe. A syringe pump (NE-1000 One Channel Programmable Syringe Pump, New Era Pump Systems, Farmingdale, NY) was used to deliver a flow rate of 2–3 mL/min through the TFF tube at 4 °C. Concentration continued until the retentate reached the intended volume (e.g., ~ 90 min to reduce volume from ~ 5 mL to ~ 0.25 mL).

For reverse dialysis, LNPs were diluted 3-fold with PBS or tris(hydroxymethyl)aminomethane buffer and added to a 3 mL Slide-A-Lyzer dialysis cassette with a 20 kDa MWCO (ThermoFisher, Waltham, MA). The LNPs were dialyzed against 15% polyethylene glycol (PEG, 35kD, Sigma-Aldrich) in RNase-free water until the sample reached the intended volume (e.g., ~ 150 min to reduce volume from ~ 2 mL to ~ 0.1 mL).

### MNP manufacturing

We considered alternative methods of screening excipients, including drying in Eppendorf tubes, but the mRNA-LNP characteristics differed significantly from mRNA-LNP reconstituted from MNPs, (Supplementary Fig. 1) so we manufactured MNPs for all experiments.

Polydimethylsiloxane (PDMS; Ellsworth Adhesives, Germantown, WI) molds were made by pouring PDMS onto metal master molds and curing overnight at 37 °C. The MNs measured 600 μm in length, 600 μm in diameter at the base, 10 μm at the tip, were mounted on a tapered pedestal measuring 300 μm in length, and were arranged in a circular array of 112 needles in an area of ~ 1 cm^2^.

LNP suspensions were mixed with an aqueous solution of PVA and sucrose to reach a final concentration of 5% w/v PVA and 10% w/v sucrose, unless otherwise noted. We observed that mixing in a 1:1 ratio of 10% w/v PVA to LNP suspension minimized patch-to-patch variability compared to a 1:3 ratio and 25% w/v PVA (Supplementary Fig. 2). A volume of 30 µL of the resulting MN cast solution was cast onto a PDMS mold while vacuum was applied from underneath to draw the MN cast solution into the MN tips. After 10 min, excess solution was wiped from the top of the mold with the flat edge of a razor blade. The MN cast was dried for 40 min under vacuum. Then, 150 µL of a backing cast solution was cast and spread to cover the MN array. The backing cast solution was dried for a minimum of 2 h on the vacuum. After drying, the patches were removed and added to a desiccator at 4 °C overnight before demolding.

The backing cast materials evaluated were SEVGILI epoxy resin (Amazon, Seattle, WA), UV-light curable glue (RapidFix, St. Louis, MO), a solution of 18% w/v polyvinyl alcohol (PVA) 4–88 (Merck, Rahway, NJ) and 18% w/v sucrose (MilliporeSigma) in RNase-free water, and a solution of 18% polyvinylpyrrolidone (PVP) K90 (MilliporeSigma) and 2% PVP K10 (MilliporeSigma) in RNase-free water.

### LNP characterization

MNPs were reconstituted by adding 200 µL of PBS onto the needle side of MNs for 90 s. The sample was gently mixed with a pipette and removed to a 0.5 mL Eppendorf tube. Complete dissolution of the MNs was confirmed by light microscopy. LNP hydrodynamic radius was measured through dynamic light scattering using a Dynapro Plate Reader (Wyatt Technology, Santa Barbara, CA). LNPs were diluted to approximately 4 µg/mL mRNA in PBS and analyzed at room temperature. Encapsulation efficiency and mRNA concentration were measured through Quant-it RiboGreen RNA Assay Kit (Invitrogen, Waltham, MA) according to manufacturer instructions [38].

### In vitro expression

RAW 246.7 cells (murine macrophage cell line, ATCC, Manassas, VA) were seeded at 10,000 cells per well in a 96-well plate and stored in an incubator at 37 °C / 5% CO_2_ for 24 h. Total mRNA concentration in each mRNA-LNP sample was measured using Quant-it RiboGreen RNA Assay Kit, for which samples were diluted in complete Dulbecco’s Modified Eagle’s Medium (90% DMEM (ATCC) and 10% heat-inactivated fetal bovine serum (ThermoFisher)) to deliver 0.1 µg of mRNA per well. After 24 h, cellular expression of luciferase protein was measured by Steady-Luc Firefly HTS Assay Kit (Biotium, Fremont, CA). For the experiment in Figs. 4, 20 µL of resuspended LNPs were added per well and the concentration was not normalized between the samples to show the effect of higher mRNA loading on in vitro expression.

### In vivo expression

All animal experiments were approved by the Georgia Institute of Technology Institutional Animal Care and Use Committee (IACUC). MNPs were pressed by thumb into the shaved backs of female BALB/c mice (3–18 months, Charles River Laboratories, Wilmington, MA) for 1 min and then taped in place for an additional 14 min. After 24 h, D-luciferin (Gold Biotechnology, St. Louis, MO) in sterile saline (75 mg/kg) was injected intraperitoneally and bioluminescent images of the mice were taken using the In Vivo Imaging System (IVIS) spectrum CT (Perkin Elmer, Shelton, CT). These images were analyzed with the IVIS software to determine the total flux of bioluminescence in photons per second (p/s) across the area of MNP insertion.

### Statistical analysis

GraphPad Prism 8 (GraphPad Software, Boston, MA) was used to generate graphs and perform statistical analyses, including Student’s t-test and one-way and two-way analysis of variance (ANOVA) with multiple comparisons. Significant differences between groups are indicated by asterisks (*p < 0.05, **p < 0.01, ***p< 0.001, ****p < 0.0001); ns indicates nonsignificant difference (*p* > 0.05).

## Results

### MNP manufacturing

**Fig. 1:**
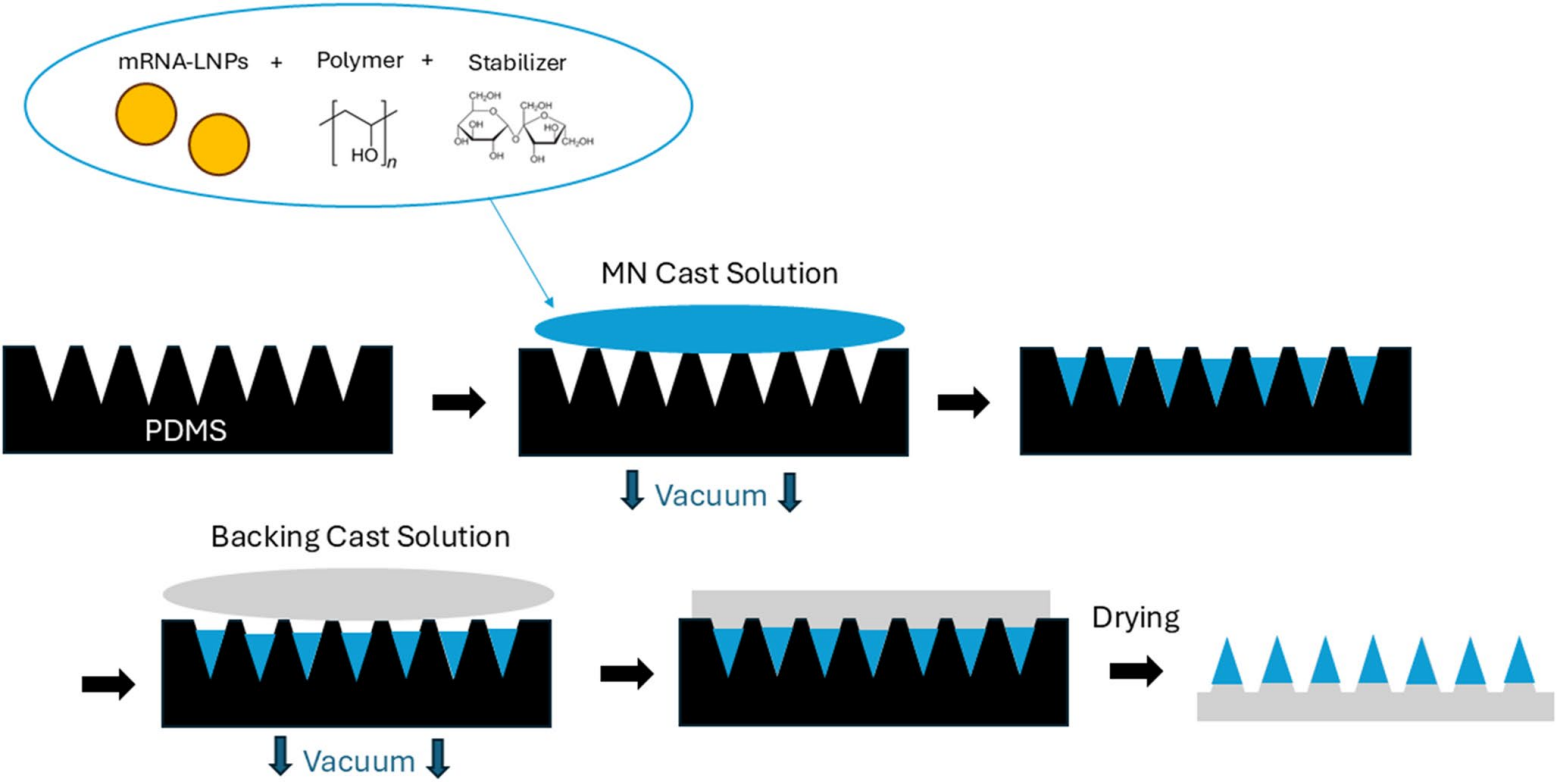
Schematic diagram of the mRNA-LNP MNP manufacturing process. A MN cast solution is cast onto a PDMS mold to fill the MN cavities of the mold with formulated mRNA-LNPs. A backing cast solution is then cast onto the mold to form the patch backing. After drying, the resulting MNP is demolded

We sought to better understand how the multi-step process of incorporating mRNA-LNPs into MNPs may impact mRNA-LNP stability (Fig. 1). mRNA-LNPs were formulated via microfluidic mixing [36], followed by formulation with excipients containing a water-soluble polymer (to form the MN matrix) and, in some cases, a stabilizer (to protect mRNA-LNP integrity) to form the MN cast solution. This solution was cast onto a PDMS mold under vacuum. After drying, a backing cast solution was prepared and cast onto the mold. This solution contained excipients without mRNA-LNPs and was used to connect the MNs and form a patch backing. After the entire MNP was dried, it was demolded for analysis (Supplementary Fig. 3). The casting solution formulation, casting process, drying process, and other components of MNP manufacturing can destabilize and damage mRNA-LNPs.

### Concentration of LNPs

The limited volume of MNPs constrains the mRNA-LNP dose that can be incorporated. While intramuscular injection can deliver up to 50 µL in mice and 2 mL in humans [39], MNPs are typically made with a MN cast solution of up to ~ 10 µL, which is about three orders of magnitude less than the typical human IM injection volume. To achieve effective mRNA dosing, mRNA-LNPs generally have to be significantly concentrated compared to conventional formulations to be incorporated into a MNP.

**Fig. 2:**
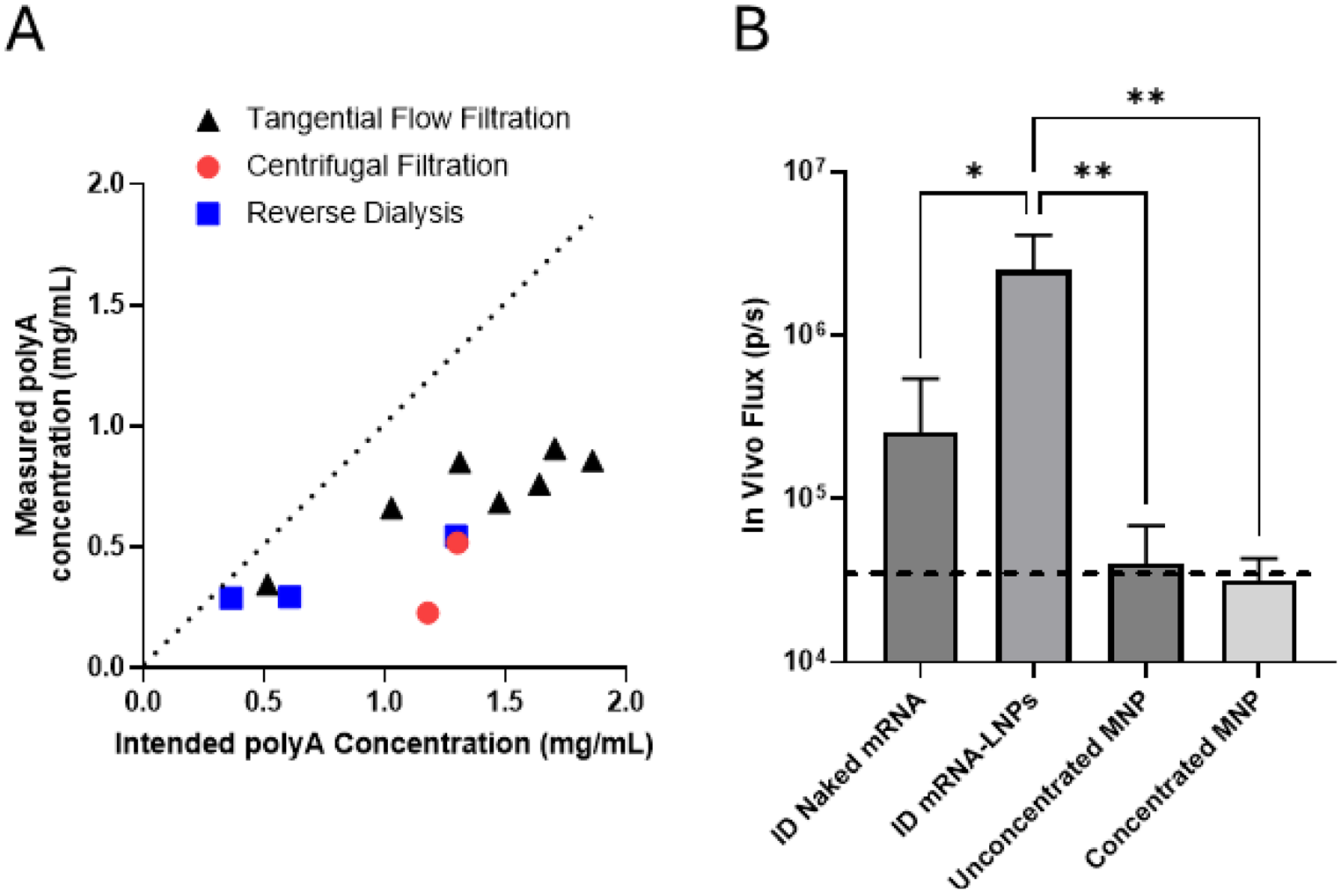
Effect of concentrating mRNA-LNPs after their fabrication. (**a**) PolyA-loaded LNPs were concentrated by TFF, centrifugal filtration, or reverse dialysis. Intended polyA concentration was determined by the change of volume of the polyA-LNP solution, while measured polyA concentration was determined by UV spectroscopy. (**b**) In vivo luminescence after delivery of luciferase-encoding mRNA-LNPs as: 1 µg naked mRNA delivered intradermally (ID), 1 µg mRNA loaded in LNPs delivered ID, 0.9 µg mRNA in unconcentrated LNPs delivered via MNP, and 1.4 µg mRNA in LNPs concentrated by TFF (7-fold by volume) and delivered by MNP. MNPs were fabricated with PVA and sucrose. The dashed line represents background luminescence levels. (*n* = 3–4, *p < 0.05, **p < 0.01, ANOVA with multiple comparisons to the ID mRNA-LNP control group)

We therefore tested three methods to concentrate mRNA-LNPs: centrifugal filtration, TFF, and reverse dialysis. For initial experiments, polyA was used as a model poly-nucleic acid as a substitute for mRNA. Though we were able to reduce liquid volume by up to 20-fold, the recovered polyA concentration in the LNP formulation was increased to a much lesser extent, indicating substantial polyA loss (Fig. 2a). Of the tested methods, TFF demonstrated least polyA loss and highest final polyA concentrations. Therefore, we chose TFF to test for in vivo application.

In control groups, intradermal (ID) injection of naked mRNA in BALB/c mice produced measurable reporter protein expression, as measured by bioluminescence measurements (Fig. 2b). ID injection of mRNA-LNPs yielded an order of magnitude greater expression, consistent with the enhanced transfection expected for LNP delivery. However, no significant luciferase expression was seen after mRNA-LNP delivery by MNP, neither for mRNA-LNPs concentrated by TFF nor for unconcentrated mRNA-LNPs. This indicates that concentrating mRNA-LNPs by TFF was not effective to enable mRNA-LNP delivery by MNP.

**Fig. 3:**
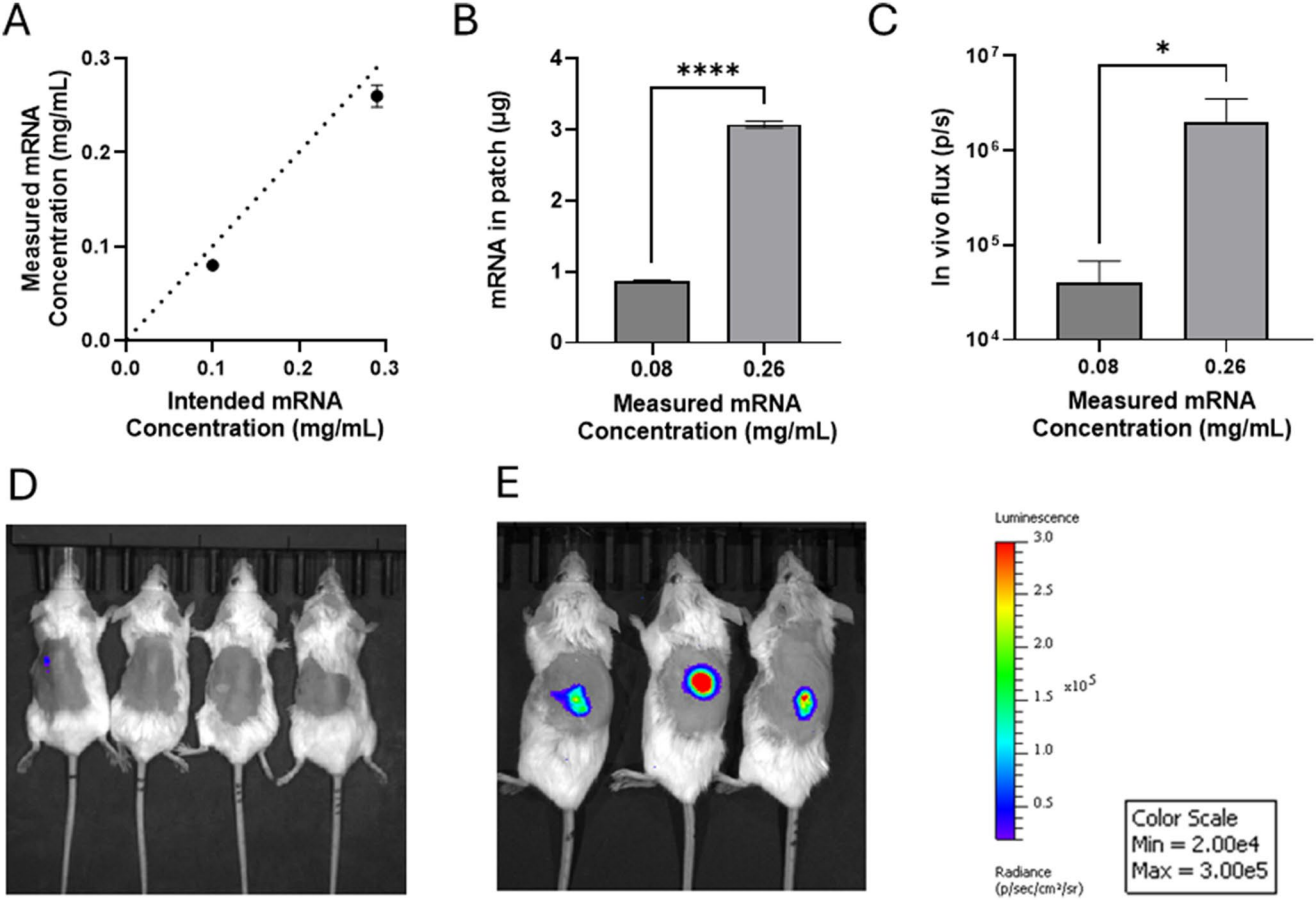
Effect of concentrating mRNA-LNPs during their fabrication. (**a**) Intended mRNA concentration (determined by change of volume of mRNA-LNP solution) compared to measured concentration (determined by UV spectroscopy). (**b**) mRNA loading in MNPs. (**c**) In vivo expression after administration of MNPs made from low-concentration (0.9 µg per patch) or high-concentration (3.0 µg per patch) luciferase-encoding mRNA-LNPs. Representative IVIS images of mice with (**d**) low-concentration mRNA-LNP MNPs or (**e**) high-concentration mRNA-LNP MNPs. (*n* = 3–4, *p < 0.05, ****p < 0.0001, Student’s t-test)

Instead of concentrating mRNA-LNPs after their fabrication, we increased the concentration of lipid and mRNA during fabrication to attain a more-concentrated mRNA-LNPs suspension. Using this approach, we increased mRNA-LNP concentration roughly three-fold (Fig. 3a), and correspondingly increased mRNA-LNP content in MNPs roughly three-fold compared to MNPs made with conventional lipids and mRNA concentrations (Fig. 3b). These higher-concentration MNPs led to significant expression in vivo (Fig. 3d-e). Notably, the three-fold increase in mRNA-LNP delivery by MNP resulted in a 93-fold increase in luciferase bioluminescence (Fig. 3c), suggesting that the increased dose exceeded the threshold for detectable protein expression [40]. We also note that these concentrated mRNA-LNPs did not undergo a buffer exchange and therefore remained at pH 3 during MNP manufacturing, a factor which is investigated in the next section.

**Fig. 4:**
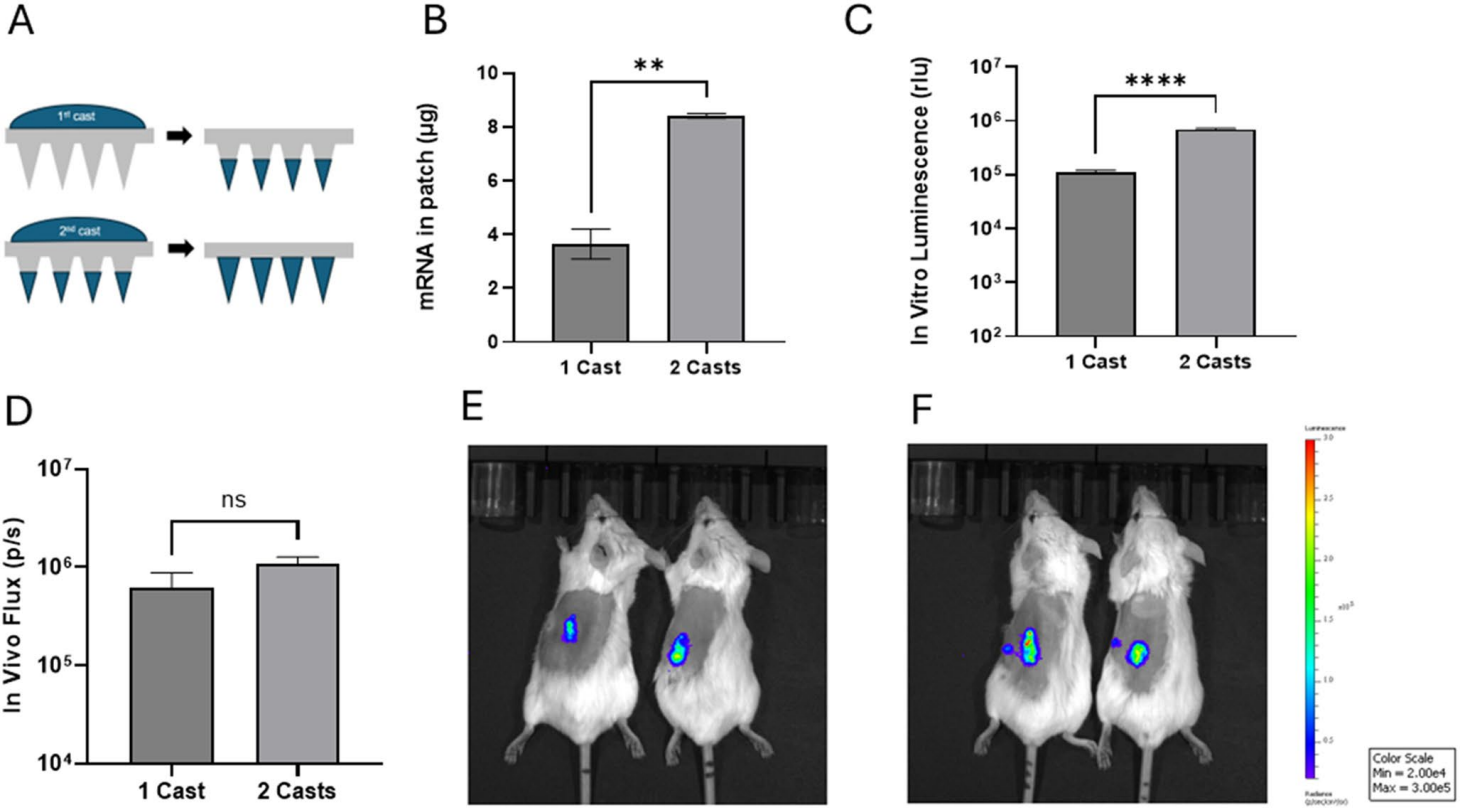
Effect of repeated casting of MN cast solution. (**a**) Schematic diagram showing the two-cast MN manufacturing approach. (**b**) mRNA loading in MNPs. (**c**) In vitro expression of luciferase-encoding mRNA-LNPs after reconstitution from MNPs. (**d**) In vivo expression of luciferase-encoding mRNA-LNPs in BALB/c mice after delivery by MNPs. The mRNA doses were 3.6 µg for one-cast and 8.4 µg for two-cast MNPs. Representative IVIS images of mice after administration of mRNA-LNP MNPs prepared with (**e**) one cast or (**f**) two casts MN cast solution. (*n* = 2, ns = no significance, **p < 0.01, ****p < 0.0001, Student’s t-test)

We wanted to further increase mRNA-LNP loading in the MNP, which we achieved by casting MN cast solution twice before adding the backing cast solution. This second cast increased the amount of mRNA-LNPs incorporated into the MNPs and filled in voids in the MNs created during drying of the first cast (Fig. 4a). This two-cast approach increased mRNA-LNP loading by roughly two-fold (Fig. 4b) and increased reporter protein expression by roughly six-fold in vitro (Fig. 4c). However, this approach did not significantly increase protein expression in vivo (Fig. 4d). Adding a third cast did not further increase mRNA-LNP loading (Supplementary Fig. 4), suggesting saturation of MN capacity. We concluded that increasing the number of sample casts was not a useful strategy for increasing mRNA expression in vivo.

### Effect of pH

While mRNA-LNPs are generally formulated at pH 3 to facilitate mRNA incorporation into LNPs by ionic bonding between negatively charged mRNA and positively charged ionizable lipids, they typically undergo subsequent buffer exchange to bring them to a physiological pH of 7.4 [11]. However, this pH change may not be needed for MNPs, since MNPs are administered in a dry state and not as a liquid injection. We hypothesized that acidic pH may be beneficial to mRNA stability by maintaining positive charge on the ionizable lipids for binding to mRNA.

We screened the effects of formulation pH between 3.0 and 7.4 after casting and drying and found that lower pH produced smaller mRNA-LNPs (Supplementary Fig. 5a), which is generally believed to improve transfection ability due to reduced aggregation and fusion [41]. pH had no significant effect on mRNA encapsulation efficiency (Supplementary Fig. 5b), which generally needs to remain high for efficient transfection [42]. We found that in vitro expression after reconstitution from a MNP was greater at low pH, while in vitro expression of non-dried LNPs appeared to be reduced at low pH (Supplementary Fig. 5c). Additionally, we found that there was no significant difference between using a phosphate or a citrate buffer when concentrating mRNA-LNPs (Supplementary Fig. 6).

**Fig. 5:**
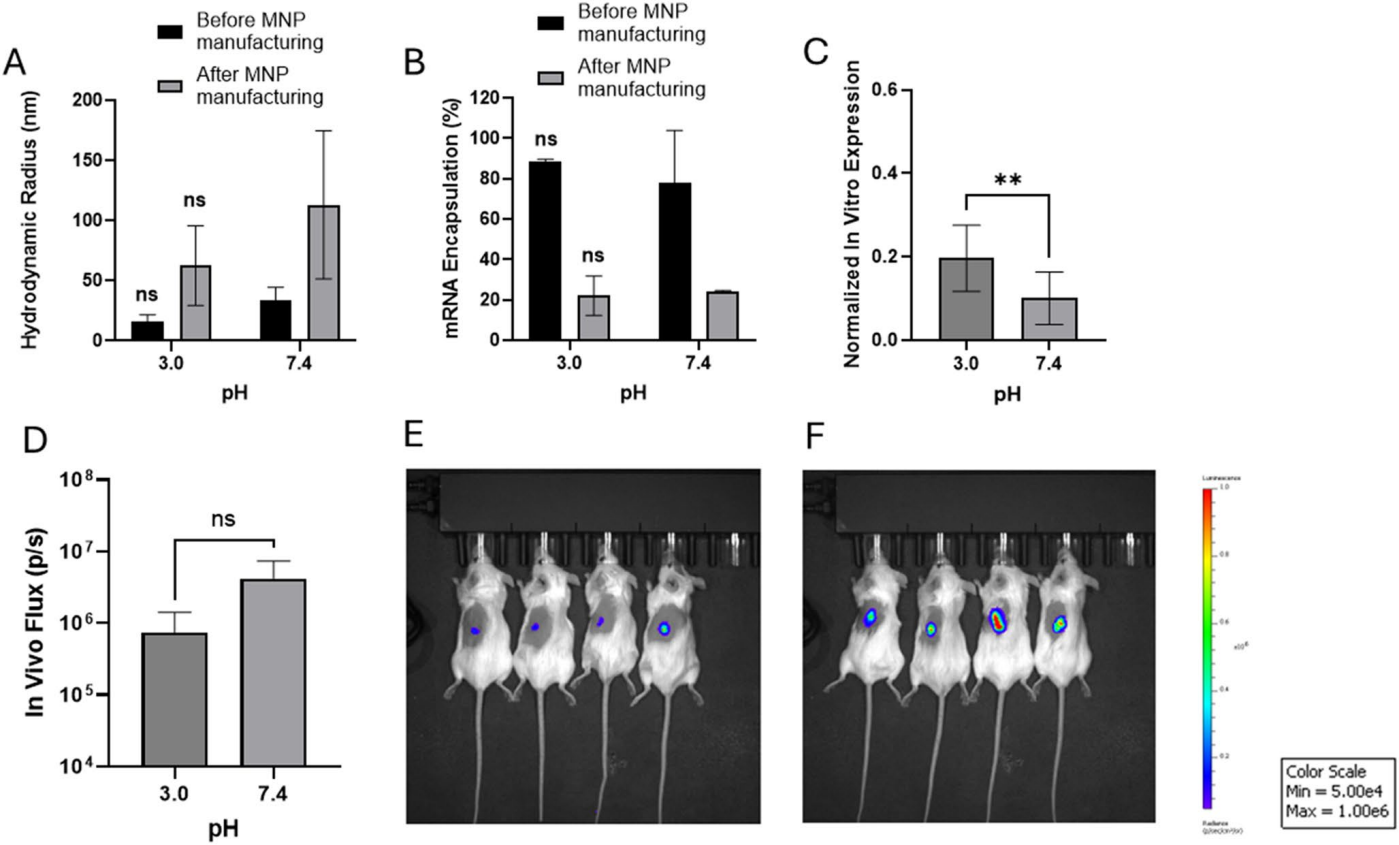
Effect of pH on mRNA-LNP stability during MNP manufacturing. (**a**) mRNA-LNP hydrodynamic radius. (**b**) mRNA encapsulation efficiency in LNPs. (**c**) In vitro cellular expression of luciferase-encoding mRNA-LNPs normalized to initial LNPs at pH 7.4. mRNA-LNPs were formulated, dialyzed in phosphate buffer at pH 3.0 or 7.4, cast onto MNPs, and reconstituted. (**d**) In vivo expression of luciferase-encoding mRNA-LNP MNPs in BALB/c mice after delivery by MNPs. Representative IVIS images of mice after application of MNPs with luciferase-encoding mRNA-LNPs at (**e**) pH 3.0 or (**f**) pH 7.4. (*n* = 4, ns = no significance, **p < 0.01, ANOVA with multiple comparisons relative to the pH 7.4 control group, Student’s t-test)

Follow-up experiments performed by keeping the mRNA-LNPs at either pH 3.0 or 7.4 throughout the MNP manufacturing process showed no significant effect of pH on mRNA-LNP size (Fig. 5a) or encapsulation efficiency (Fig. 5b), significantly better in vitro expression at pH 3.0 (Fig. 5c) and no significant difference in in vivo expression (Fig. 5d). Mice observed for several days after application of MNPs showed no signs of reactogenicity or ulceration at the MNP application site at either pH. We concluded that there was no clear advantage to either low or high pH to mRNA-LNP effectiveness but favored use of low pH since it simplified manufacturing by eliminating the buffer-exchange step.

### Effect of MNP formulation excipients

**Fig. 6:**
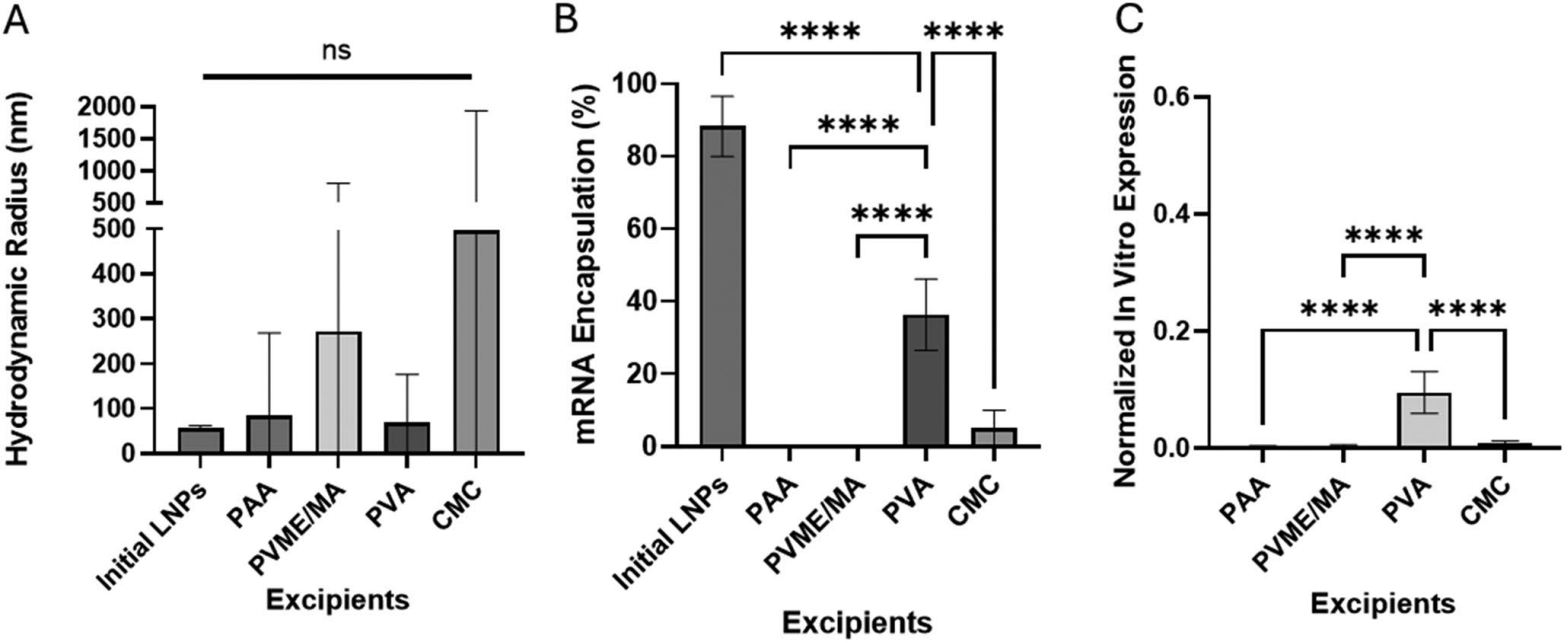
Effect of polymeric MNP formulation excipients. (**a**) mRNA-LNP hydrodynamic radius. (**b**) mRNA encapsulation efficiency in LNPs. (**c**) In vitro cellular expression of luciferase-encoding mRNA-LNPs normalized to initial LNPs. mRNA-LNPs were evaluated after reconstitution from MNPs formulated with polyacrylic acid (PAA), poly(vinyl methyl ether-co-maleic acid) (PVME/MA), polyvinyl alcohol (PVA), or carboxymethyl-cellulose (CMC). (*n* = 6, ns = no significance, ****p < 0.0001, ANOVA with multiple comparisons relative to the PVA control group)

Water-soluble polymers are used to stabilize excipients and provide mechanical strength to MNPs [43]. Our initial comparison of MN cast solutions containing PVA and PVP demonstrated that PVA preserved mRNA-LNP size after MNP manufacturing (Supplementary Fig. 7). Further screening against polyacrylic acid (PAA), poly(vinyl methyl ether-co-maleic acid) (PVME/MA), and carboxymethyl-cellulose (CMC) across multiple measures of LNP stability demonstrated PVA’s superiority. MNPs manufactured with PVA maintained a small size, greater encapsulation, and greater in vitro cellular expression than the other formulations (Fig. 6). We therefore used the PVA-based MN cast solution formulation in all other experiments.

We also studied the effects of PVA concentration and observed that higher PVA content increased in vitro cellular expression. Low PVA content (1% or 5%) maintained smaller mRNA-LNP size after MNP manufacturing (Supplementary Fig. 8a) and any amount of PVA decreased mRNA encapsulation in LNPs (Supplementary Fig. 8b). However, the in vitro expression post MNP manufacturing exponentially increased with increasing PVA content (Supplementary Fig. 8c). We tested the effect of PVA on mRNA-LNPs before MNP manufacturing and found that PVA content increased in vitro expression only in the presence of sucrose (Supplementary Fig. 8d). From these data, we concluded that PVA has a positive effect on mRNA-LNP transfection beyond its protective effects during the MNP manufacturing process, but further research is needed to determine whether this offers translational potential.

**Fig. 7:**
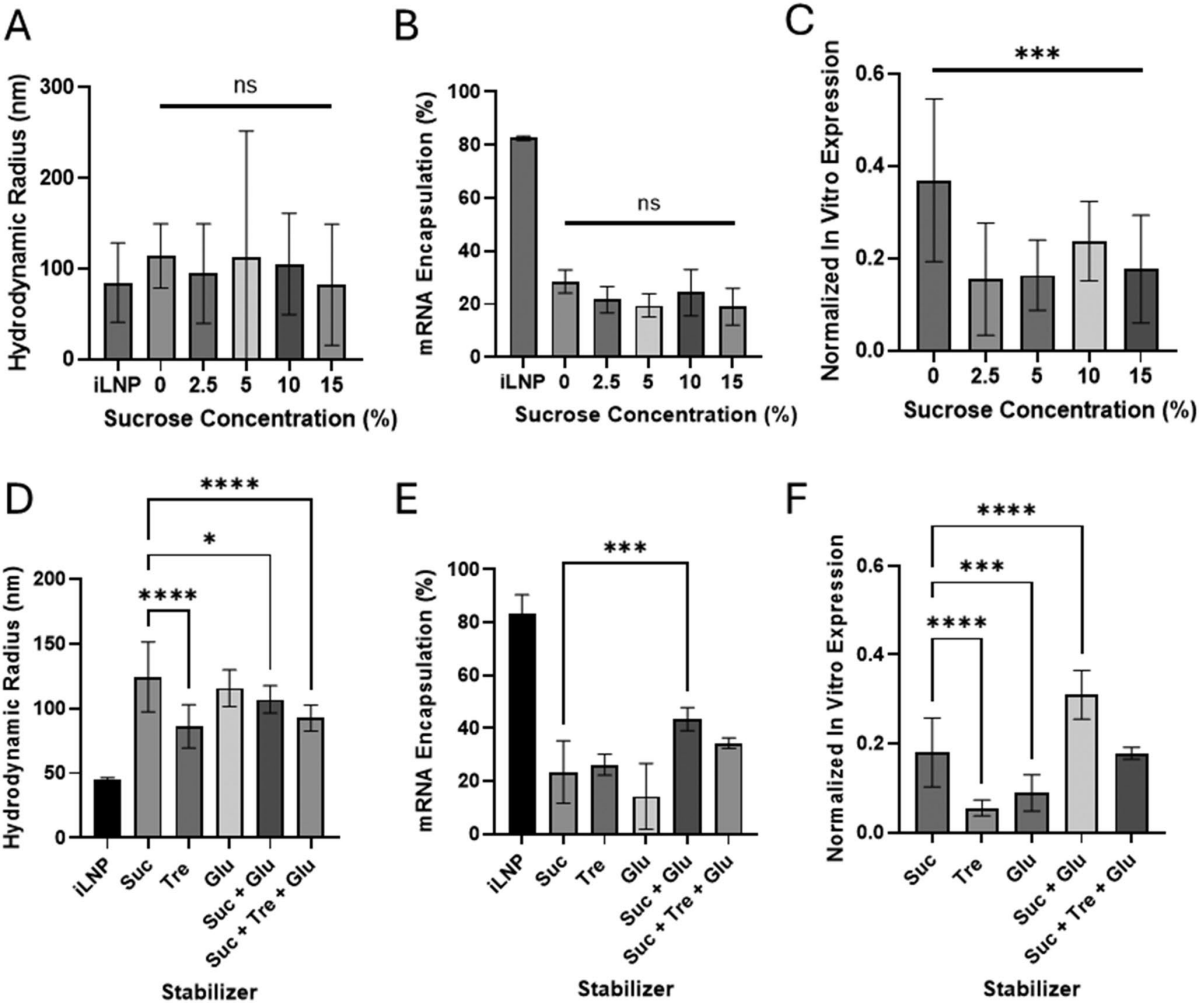
Effect of sugar-based MNP formulation excipients. (**a**, **d**) mRNA-LNP hydrodynamic radius. (**b**, **e**) mRNA encapsulation efficiency in LNPs. (**c**, **f**) In vitro cellular expression of luciferase-encoding mRNA-LNPs normalized to initial LNPs. mRNA-LNPs were evaluated after reconstitution from MNPs formulated with 5% PVA and (**a**-**c**) varying concentrations of sucrose, or (**d**-**f**) 10% sucrose (Suc), trehalose (Tre), glucose (Glu), sucrose/glucose, or sucrose/glucose/trehalose. (*n* = 6, ns = no significance, *p < 0.05, **p < 0.01, ***p < 0.001, ****p < 0.0001, ANOVA with multiple comparisons relative to the 0% control group and sucrose control group)

In addition to water-soluble polymers, other excipients may be added to a MNP to increase stability, often including sugars [19]. We screened two disaccharides (sucrose and trehalose) and a monosaccharide (glucose) to assess their effects of mRNA-LNP stability [17, 44] We found that addition of sucrose to the MN cast solution over a range of concentrations did not significantly affect mRNA-LNP size (Fig. 7a) or encapsulation (Fig. 7b) after MNP manufacturing, but did generally decrease in vitro expression (Fig. 7c). Formulation with trehalose, glucose, and combinations of two or three sugars indicated that trehalose helped maintain smaller mRNA-LNP size (Fig. 7d). Combination of sucrose and glucose maintained smaller mRNA-LNP size (Fig. 7d), greater encapsulation efficiency (Fig. 7e) and increased in vitro cellular expression (Fig. 7f). However, all of these improvements were modest, indicating that additional sugars provided minimal benefit beyond the stabilization already conferred by PVA. However, additional exploration into other stabilizers may further improve mRNA-LNP MNPs.

### Effect of MNP drying conditions

Drying process represents a critical stress factor for lipid-based nanoparticles, and previous studies on air-drying of liposomes indicate that slowing the drying process can increase stability [45]. Additionally, lower temperatures generally slow degradation processes for LNPs and mRNA [46, 47]. Therefore, we hypothesized that maintaining a lower temperature (i.e., which would also slow the drying process) during the MNP manufacturing process would improve stability. We manufactured MNPs by casting the MN casting solution and backing cast solution at 25 °C, and then storing the MNPs with desiccant for two days at 5 °C, 25 °C, or 40 °C.

**Fig. 8:**
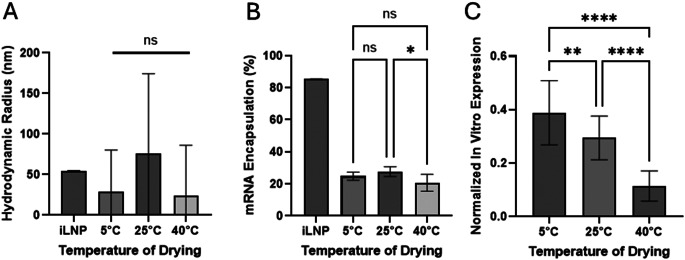
Effect of drying temperature during MNP manufacturing. (**a**) mRNA-LNP hydrodynamic radius, (**b**) mRNA encapsulation efficiency in LNPs, and (**c**) in vitro cellular expression of luciferase-encoding mRNA normalized to initial LNPs as a function of MNP drying temperature. mRNA-LNPs were evaluated after reconstitution from an MNP formulated with PVA-sucrose, backed with epoxy, and stored for two days with desiccant in a controlled-temperature stability chamber. (*n* = 6, ns = no significance, *p < 0.05, **p < 0.01, ****p < 0.0001, ANOVA with multiple comparisons between all groups after MNP manufacturing)

The drying temperature had no significant effect on mRNA-LNP size (Fig. 8a). There was a slight reduction in encapsulation efficiency at 40 °C (Fig. 8b). In contrast, the in vitro expression showed a significant correlation between lower drying temperature and greater mRNA expression (Fig. 8c). We concluded that drying at a low temperature favored mRNA-LNP stability. This discrepancy between physical characteristics and functional activity points to a factor other than LNP integrity, such as mRNA integrity, that might be compromised by higher temperatures.

**Fig. 9:**
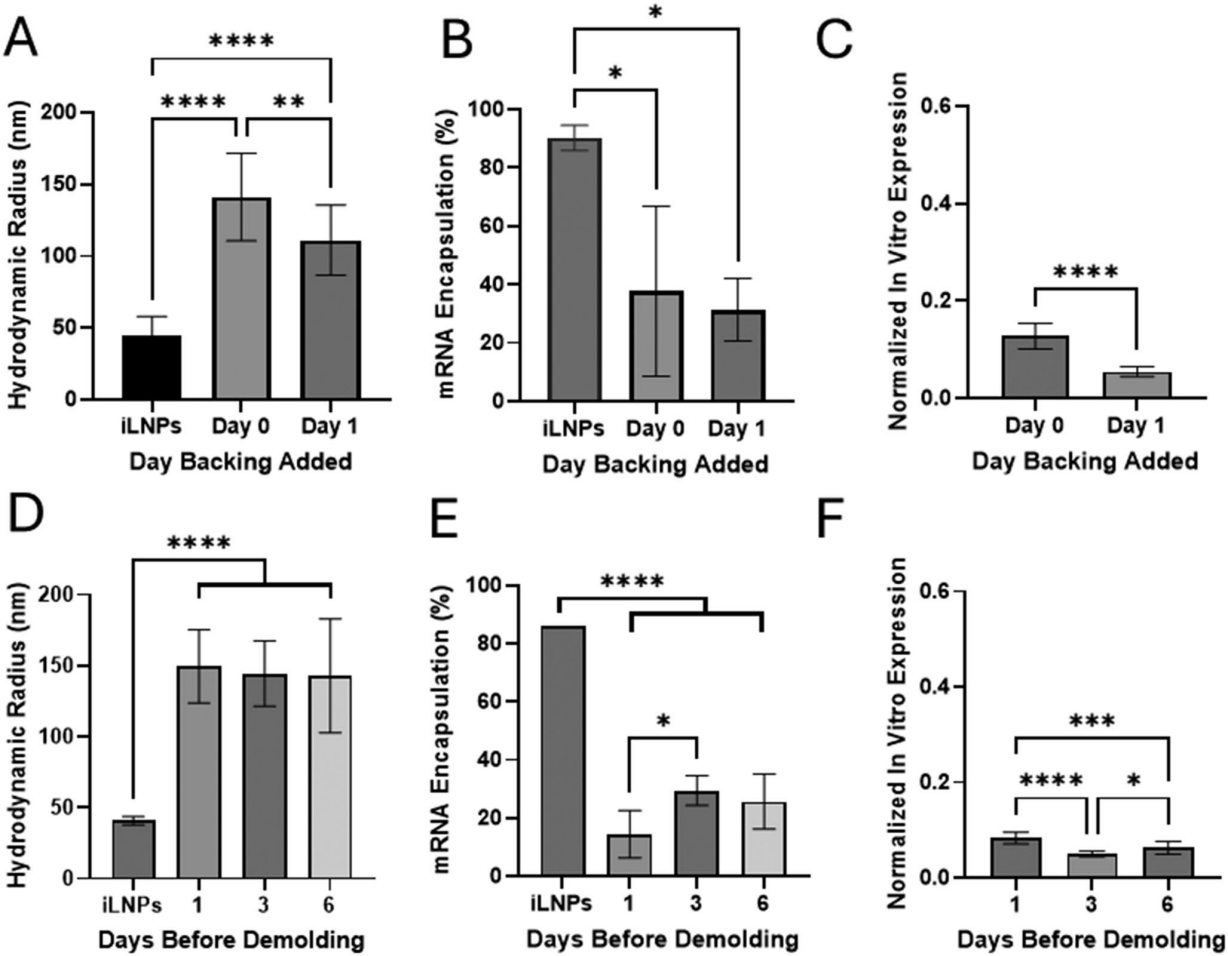
Effect of drying conditions on mRNA-LNP stability in an MNP. (**a**-**c**) Effect of adding the backing cast solution on the same day (Day 0) or after drying the sample cast overnight (Day 1). (**d**-**f**) Effect of the length of the drying process time. Effects of drying conditions were assessed by measuring (**a**, **d**) hydrodynamic radius of mRNA-LNPs, (**b**, **e**) mRNA encapsulation in an MNP, and (**c**, **f**) in vitro cellular expression of luciferase-encoding mRNA-LNPs normalized to initial LNPs. (*n* = 6, *p < 0.05, **p < 0.01, ***p < 0.001, ****p < 0.0001, ANOVA with multiple comparisons between all groups, Student’s t-test)

We also investigated the effects of drying time on mRNA-LNP stability. First, we measured the effects of adding the backing cast solution on the same day as the sample cast solution (before complete drying of the sample cast occurred) and compared with more-fully drying the sample cast overnight before adding the backing cast solution. When the sample cast was dried overnight, the mRNA-LNPs had a smaller size (Fig. 9a), a similar encapsulation efficiency (Fig. 9b), and a lower in vitro expression (Fig. 9c). We concluded that it was beneficial to add the backing cast solution on the same day rather than more-fully drying the sample cast overnight. We hypothesized that MNPs in the mold dried slowly and, to understand this, we studied the impact of the time the MNPs remained in the PDMS mold before demolding. When varying the time of MNP demolding between one and six days, we found that the demolding time had no significant effect on mRNA-LNP size (Fig. 9d). The encapsulation efficiency was higher in MNPs that were demolded after more than one day (Fig. 9e). The cellular expression was highest from the MNPs demolded after one day, but the effect was minimal (Fig. 9f). We concluded that there was no clear advantage to waiting more than one day to demold the MNPs.

### Effect of MNP backing cast formulation

After loading the MNs with the sample cast, we added a backing cast solution to form the rest of the MNP and provide mechanical strength for insertion. We tested the effects of the backing cast solution on the mRNA-LNPs during MNP manufacturing. We screened four different backings solutions: a self-setting epoxy, ultraviolet (UV)-curable glue, an aqueous solution of PVA and sucrose, and an aqueous solution of PVP. We also tested epoxy backing with exposure to UV light to evaluate the effect of UV light exposure independent of its use to cure the UV-curable glue.

**Fig. 10:**
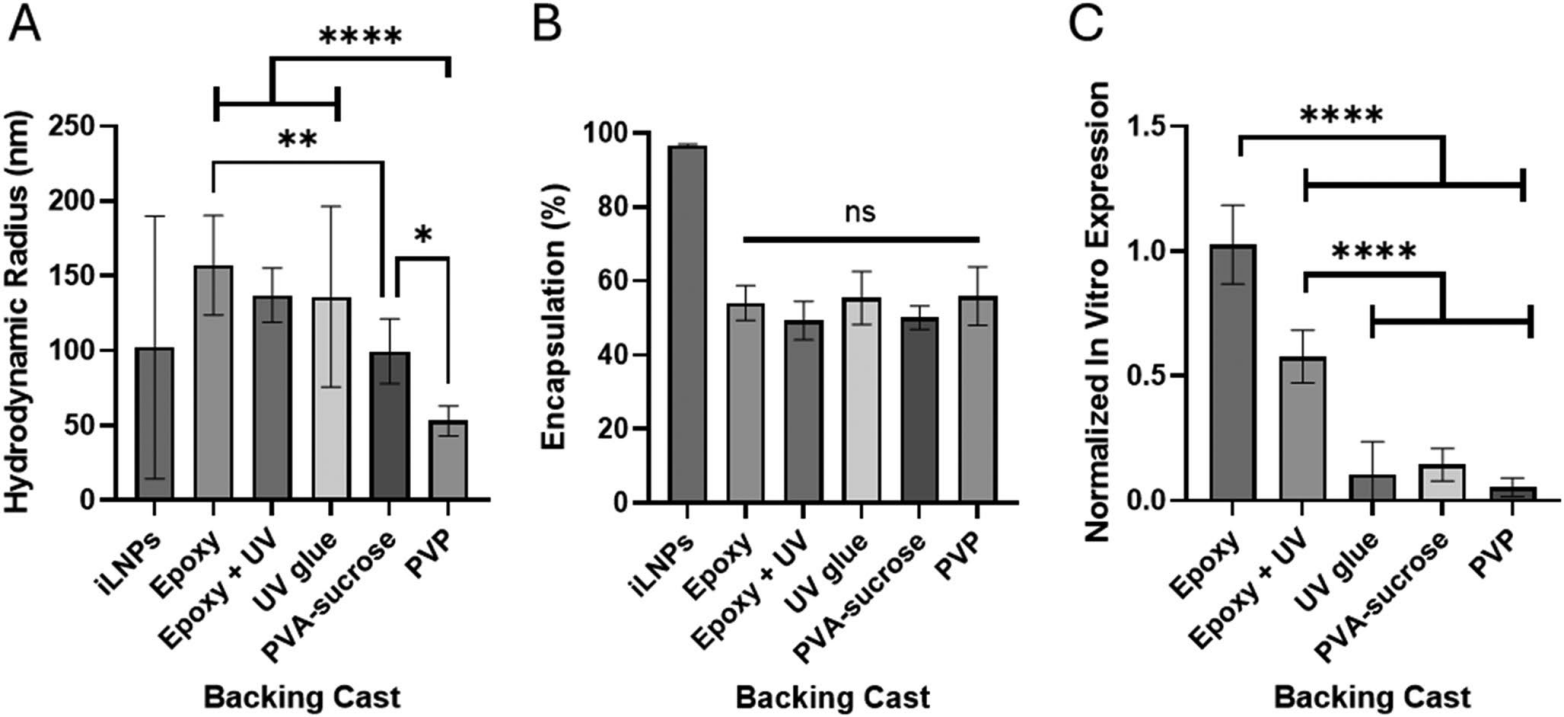
Effect of MNP backing cast solutions. (**a**) mRNA-LNP hydrodynamic radius, (**b**) mRNA encapsulation efficiency, and (**c**) in vitro cellular expression of luciferase-encoding mRNA-LNPs normalized to initial LNPs shown as a function of backing cast solution. mRNA-LNPs were evaluated after reconstitution from a MNP formulated with PVA-sucrose and backed with epoxy, epoxy with exposure to UV light, UV-curable glue and exposure to UV light, an aqueous solution 18% w/v PVA and 18% w/v sucrose, or 20% w/v PVP. (*n* = 4, ns = no significance, *p < 0.05, **p < 0.01, ****p < 0.0001, ANOVA with multiple comparisons between all groups after MNP manufacturing)

The choice of backing cast solution had a significant effect on mRNA-LNP size, with PVA-sucrose and PVP casting solution resulting in the smallest mRNA-LNPs (Fig. 10a), and no significant effect on mRNA encapsulation (Fig. 10b). The epoxy backing resulted in the highest in vitro expression with and without UV light, but exposure to UV light roughly halved the expression (Fig. 10c). An additional factor to consider was that the backings required different drying/curing times, which affected the overall manufacturing time of the MNP. Aqueous backings, such as PVA-sucrose and PVP, took up to seven days to dry at refrigeration temperatures. The epoxy backing took less than one day to fully cure. The UV-curable glue cured within 30 s during exposure to UV light. Because of the stability benefit and relatively short manufacturing time, we concluded that epoxy was the best backing cast solution.

### Thermostability of mRNA-LNPs in MNPs

**Fig. 11:**
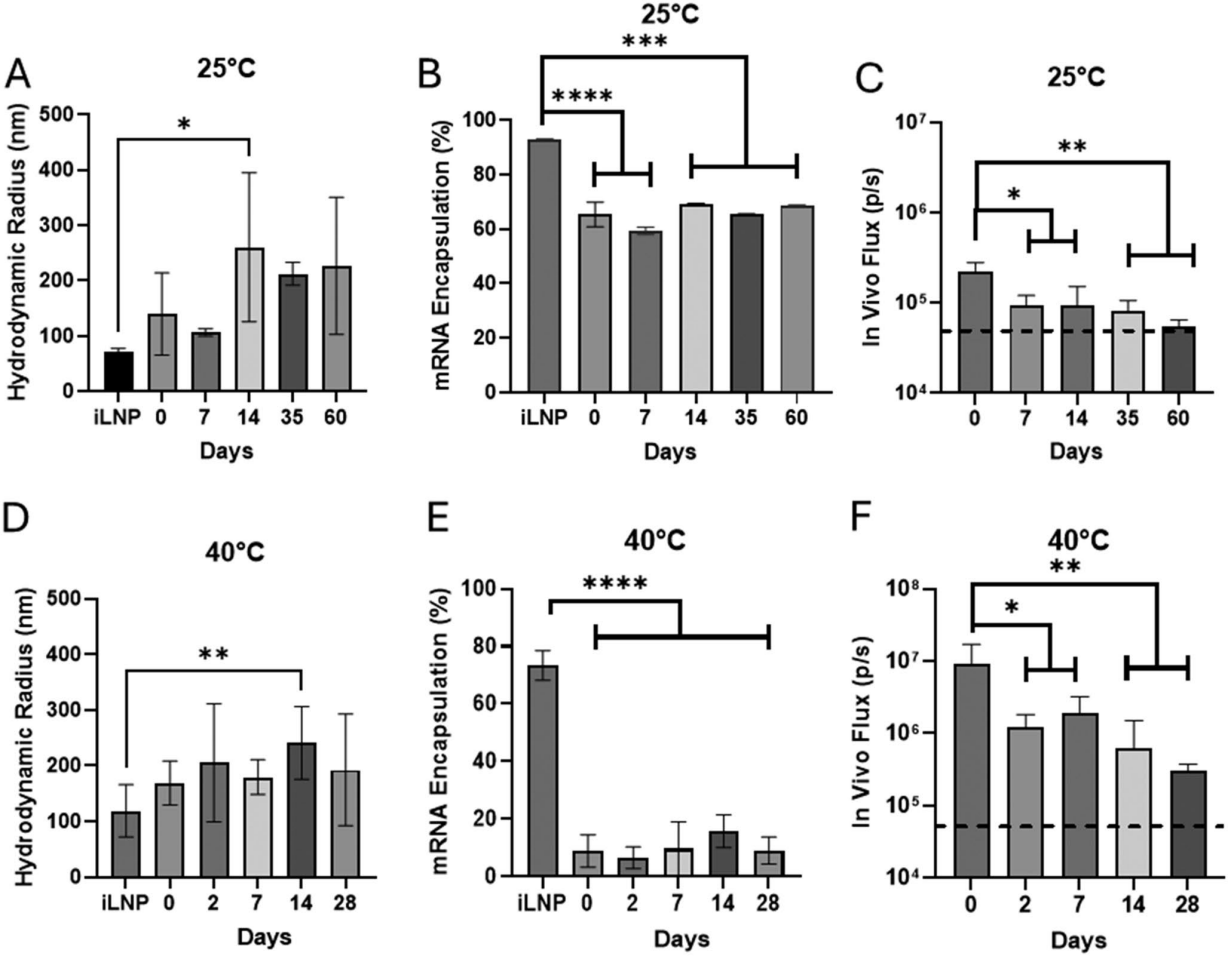
Thermostability of mRNA-LNP MNPs. (**a**-**c**) Thermostability of mRNA-LNP MNPs at 25 °C over 60 days, as measured by (a) mRNA-LNP hydrodynamic radius, (**b**) mRNA encapsulation efficiency in LNPs, and (**c**) in vivo expression of luciferase-encoding mRNA-LNP MNPs in mice. (**d**-**f**) Thermostability of luciferase-encoding mRNA-LNP MNPs at 40 °C over 28 days, as measured by (**d**) mRNA-LNP hydrodynamic radius, (**e**) mRNA encapsulation efficiency in LNPs, and (**f**) in vivo expression of luciferase-encoding mRNA-LNP MNPs in mice. The dashed lines represent background levels of luminescence. (*n* = 2–6, *p < 0.05, **p < 0.01, ***p < 0.001, ****p < 0.0001, ANOVA with multiple comparisons to control group at Day 0)

In addition to assessing mRNA-LNP stability during MNP manufacturing, we also tested the thermostability at 25 °C for 2 months and 40 °C for one month. At both temperatures, mRNA-LNP size (Fig. 11a and d) and encapsulation (Fig. 11b and e) did not change significantly between day 0 and the end of the study. At 25 °C, in vivo expression dropped by 59% in the first week and then remained just above background levels without significantly decreasing after day 7 for up to 60 days (*p* = 0.50, Fig. 11c). At 40 °C, in vivo expression dropped by 87% in the first two days and then continued to decrease out to 28 days, ending with a cumulative drop of 97% (*p* = 0.02, Fig. 11f). We concluded that mRNA-LNP MNPs lost activity during storage, and the most significant loss in mRNA expression occurred within the first week.

## Discussion

### LNP concentration

We concentrated mRNA-LNPs to increase their loading into an MNP. Among the three concentration steps studied, TFF demonstrated better results and would also be the easiest to scale up. However, mRNA-LNPs concentrated by TFF did not yield in vivo expression, which likely resulted from mRNA-LNP loss and destabilization during the lengthy concentration steps.

As an alternative to concentrating LNPs after manufacturing, LNPs were formulated at higher concentrations to yield 0.29 mg/mL of mRNA in LNPs. After delivering these mRNA-LNPs via MNP without further concentration, in vivo expression was achieved. We concluded that minimizing the manufacturing steps led to greater expression. Due to lipid solubility limits and the volumes required to formulate LNPs, there is an upper limit to the mRNA concentration possible with this method, so further work will be needed to improve the mRNA loading beyond approximately 3 µg of mRNA per MNP.

Casting the MN cast solution multiple times during MNP manufacturing can increase mRNA-LNP loading in a MNP without increasing the concentration of the cast solution. Two casts improved mRNA loading, but this did not translate to a significant increase of in vivo expression, which could be explained by incomplete delivery into the skin. Future work should include starting from high-concentration LNPs, followed by a concentration step such as TFF, and testing additional MNP designs to enable loading of larger doses of mRNA.

### Effect of pH

We explored the effects of pH on mRNA-LNP stability during MNP manufacturing. Of note, in vitro data supported the use of a lower pH during MNP manufacturing, but in vivo data did not favor either pH. This points to differences in in vitro and in vivo expression of mRNA-LNPs, an observation made by other groups as well [48]. Further studies should consider the effects of pH, but the results of this study support minimizing the processing steps for MNP manufacturing, which may involve leaving mRNA-LNPs at pH 3.

### Excipients

We screened several polymers for their stabilizing effects on mRNA-LNPs during MNP manufacturing. Of the polymers tested, PVA was the most stabilizing, as demonstrated by a small hydrodynamic radius, a higher mRNA encapsulation, and a better in vitro expression compared to the other polymers. This could be explained through the water replacement hypothesis, which suggests that LNPs in suspension rely on interactions with water molecules to stabilize their structure [41, 49]. An excipient that can replace water during the drying process through hydrophilic interactions or hydrogen bonding with the LNPs will increase stability. PVA contains hydrophilic hydroxyl groups that could act as a replacement for water in this context, stabilizing the mRNA-LNPs during drying. Other tested polymers, such as CMC, contain hydroxyl groups, but these polymers have more complex structures. We hypothesize that steric hindrance may have reduced their efficacy as stabilizers. Despite their role as stabilizers during other drying operations [47, 50], sucrose, trehalose and glucose did not further improve stability when PVA was used for manufacturing MNPs. More research is needed to explain the role of PVA and other materials in stabilizing LNPs during drying.

### Drying temperature

We observed that a lower temperature (5 °C) during MNP drying preserved mRNA-LNP function after MNP manufacturing. This may have been due to the slower drying or slower reaction kinetics of degradation processes. The results support storing mRNA-LNP MNPs in a refrigerated environment during the drying step of manufacturing.

### Backing material

The backing cast solution was added to form the non-drug-containing parts of the MNP and enhance mechanical strength. However, we observed that the backing cast solution could have a significant effect on in vitro expression of mRNA-LNPs after MNP manufacturing. We hypothesized that these effects were due to the drying rate of the entire MNP, exposure to solvents or manufacturing conditions, or rewetting the first cast [51]. Rewetting occurred when the second cast was an aqueous solution that could dissolve the dried components of the MN cast solution and subject them to a second dissolution and drying process. In addition to rewetting, exposures to the UV light that was used to cure the UV-curable glue appeared to harm the mRNA. Overall, the epoxy backing yielded the best results, presumably because it did not mix with the sample cast and did not require exposure to UV light. These findings indicate that the backing cast is an important consideration when designing a mRNA-LNP MNP.

### Thermostability

mRNA-LNP vaccines have been stored for a month at refrigeration temperatures (2–8 °C) or a week at room temperature before losing potency [52]. MNPs have been shown to improve stability for other vaccine types, so there is interest in their ability to similarly stabilize mRNA vaccines [53]. In this study, mRNA-LNP MNPs produced measurable in vivo expression after storage at 40 °C for 28 days, but the expression level was significantly reduced from its initial value.

### Limitations and future work

This study had limitations, such as testing only certain MNP formulation and manufacturing factors over certain ranges, such that a complete optimization was not possible; studying outcomes in some cases only in vitro, and not in vivo; and using sample sizes that were relatively small. While mRNA-LNP size was generally in the range known to be effective for transfection, mRNA encapsulation efficiency was sometimes low. Additional characterization of the mRNA-LNPs, such as measuring zeta potential and polydispersity before and after MNP manufacturing could provide additional insight into interactions between the mRNA-LNPs and the polymer matrix. Future studies should use mRNA encoding therapeutic proteins or vaccines and test their safety and efficacy in vivo.

Despite optimization, loss of mRNA encapsulation was a persistent problem during MNP manufacturing, which we attribute to the drying process. While a high encapsulation efficiency is generally believed to be associated with high transfection rates [1], our data did not always show such a relationship. Further developments to address the loss of encapsulation efficiency could involve (1) improving formulation using stabilizers beyond water-soluble polymers and sugars; (2) identifying the role of the lipid composition in LNP stability during the MNP manufacturing process to design a more stable LNP; and (3) improving MNP drying by adjusting the environmental humidity or the composition of the air. We hypothesize that improvements to the encapsulation efficiency can play a meaningful role in improving mRNA expression after incorporation into an MNP.

## Conclusions

The development of a MNP for delivering mRNA-LNPs involved investigation into multiple aspects of the MNP formulation and manufacturing process, including mRNA-LNP concentration steps, formulation excipients, manufacturing conditions, and backing cast solutions. mRNA-LNPs that were fabricated at a higher concentration maintained stability during MNP manufacturing more effectively than mRNA-LNPs that were concentrated after mRNA-LNP fabrication. Among the tested factors, polymer choice had the strongest effect on LNP stability, and PVA was the most stabilizing polymer. Additional stabilizers, including sucrose, trehalose, and glucose, offered little or no improvement over PVA alone. Low manufacturing temperature (5 °C) helped preserve mRNA-LNP function. The MNP backing cast played a role in stability, and an epoxy backing maintained the highest mRNA-LNP expression. Finally, MNPs that were stored at 25–40 °C maintained in vivo expression for several weeks, although at reduced levels over time. The findings from this study can be used to further understand mRNA-LNP technology and aid in the development of MNP delivery methods.

## Supplementary Information

Below is the link to the electronic supplementary material.

Supplementary Material 1

## Data Availability

Data available by contacting the authors.
