# Supplementary material for: Development of a microneedle patch for delivery of mRNA-lipid nanoparticles
Source: Drug Deliv Transl Res. 2025 Sep 2;16(7):2256–71. doi: 10.1007/s13346-025-01964-z (PMC13294314; doi:10.1007/s13346-025-01964-z)
Supplement: Supplementary file 1 — Supplementary Material 1 [file 13346_2025_1964_MOESM1_ESM.docx]

SUPPLEMENTARY INFORMATION

**Development of a Microneedle Patch for Delivery of mRNA-Lipid Nanoparticles**

Sophia H. Sakers^1^, B. Pradeep K. Reddy^2^, Gianna Fiduccia^2^, Katherine Byrne^1^, Ingrid Stén^2,3^, Julie Kim^1^, Afsane Radmand^2^, James E. Dahlman^1^, Mark R. Prausnitz^1,2^

^1^Wallace H. Coulter Department of Biomedical Engineering, Georgia Institute of Technology and Emory University School of Medicine, Atlanta, GA, USA

^2^School of Chemical and Biomolecular Engineering, Georgia Institute of Technology, Atlanta, GA, USA

^3^KTH Royal Institute of Technology, Stockholm, Sweden


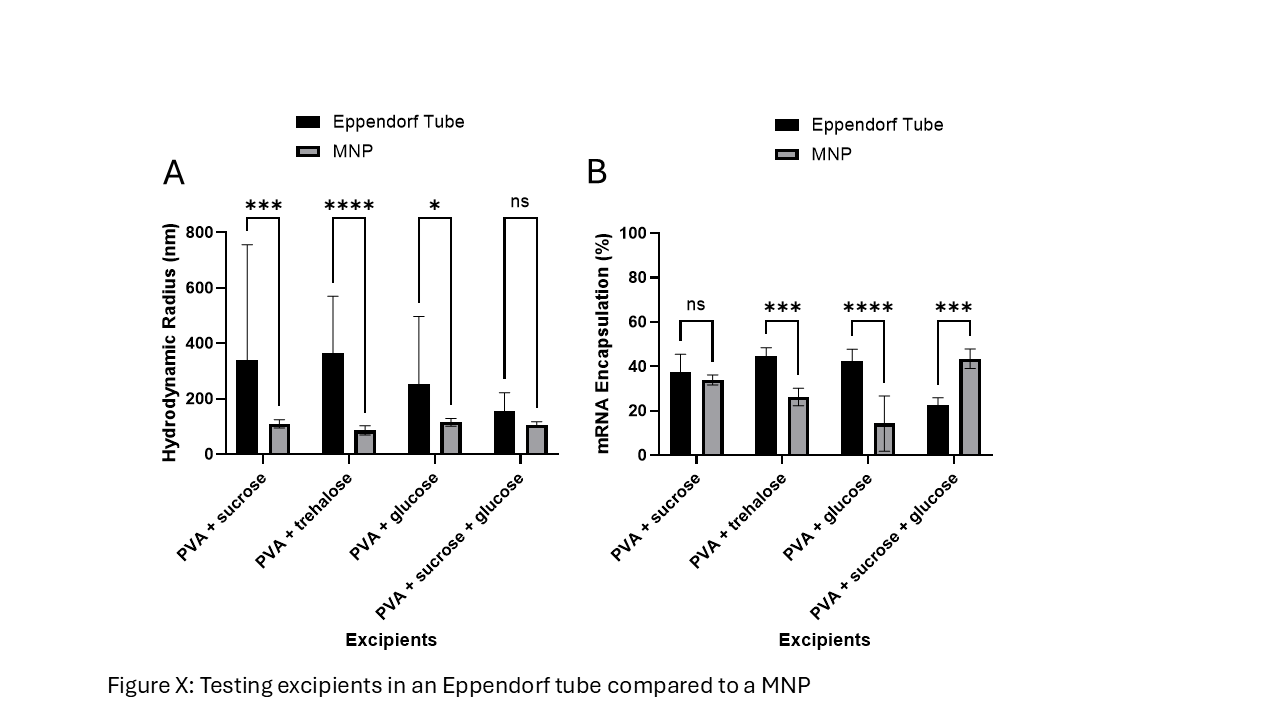


Supplementary Fig. 1: Effect of drying in an Eppendorf tube compared to a MNP on (a) hydrodynamic radius of a mRNA-LNP and (b) mRNA encapsulation in a LNP. Significant differences were observed between the two drying methods. (n=3-6, ns=no significance, *p<0.05, ***p<0.001, ****p<0.0001, two-way ANOVA)


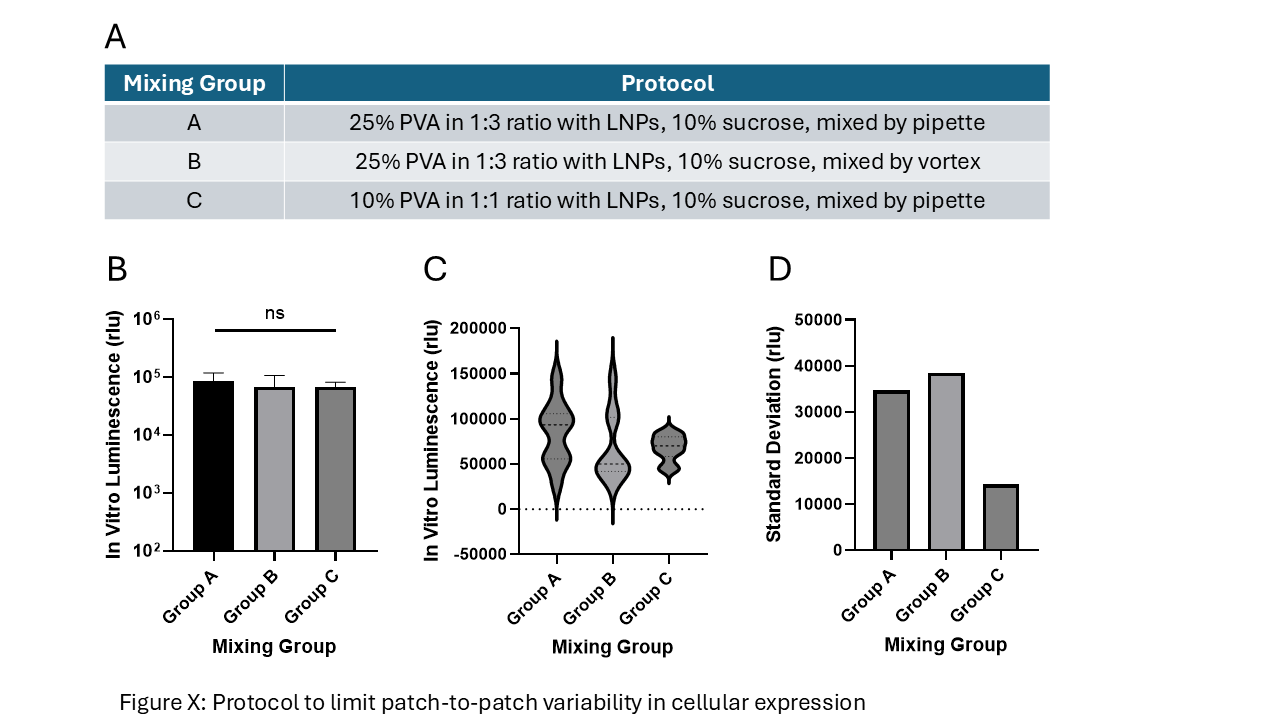


Supplementary Fig. 2: Study to correct patch-to-patch variability among mRNA-LNP MNPs. We used 25% PVA in a 1:3 ratio with LNPs to improve mRNA loading, but we noticed significant patch-to-patch variability, so we hypothesized that the sample cast was non-homogenous and tested alternative mixing methods*.* (a) Table of the mixing groups. (b, c) In vitro expression of luciferase-encoding mRNA-LNPs. (d) Standard deviation of in vitro expression. (n=6, ns=no significance, one-way ANOVA)


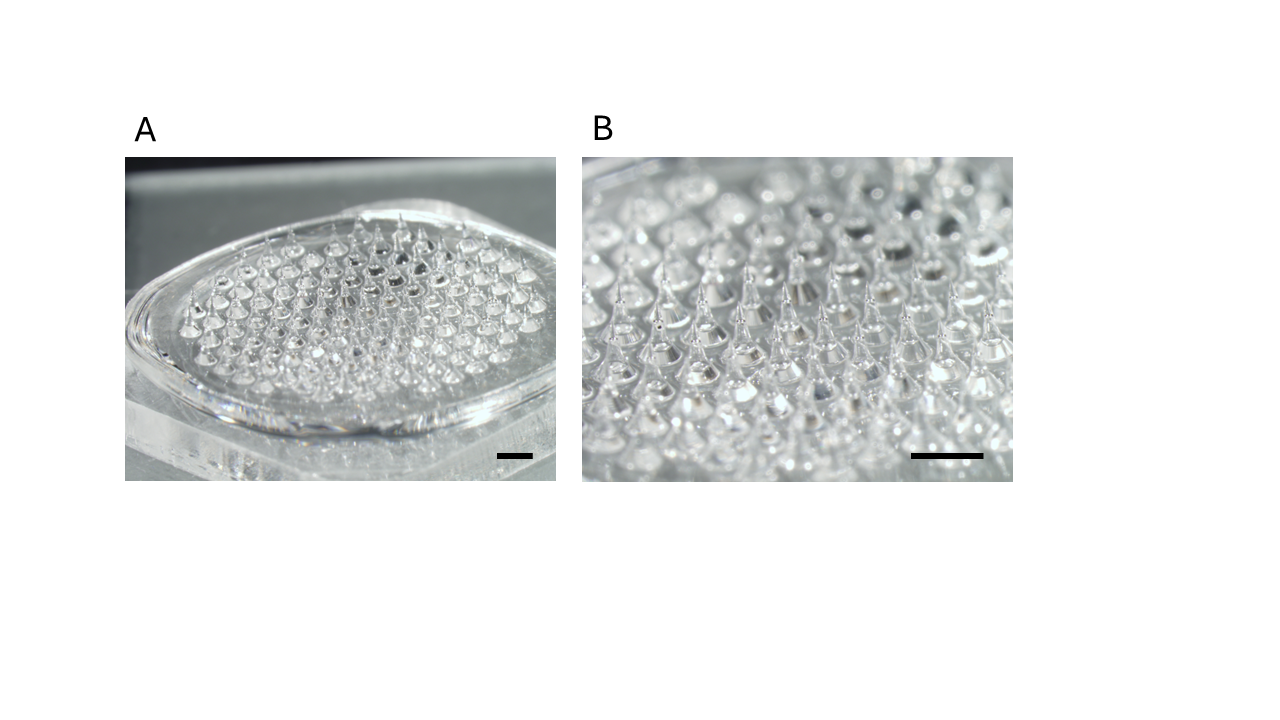


**Supplementary Fig. 3**: Representative light microscopy images of a MNP. Scale bars: 1 mm. Microscopy images taken by SZX-ILLB2-100 Olympus Microscope.


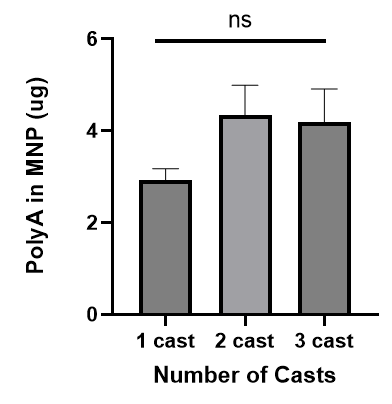


Supplementary Fig. 4: Effect of repeated casting of MN cast solution on loading of polyA in LNPs. (n = 2-3, ns = no significance, one-way ANOVA)


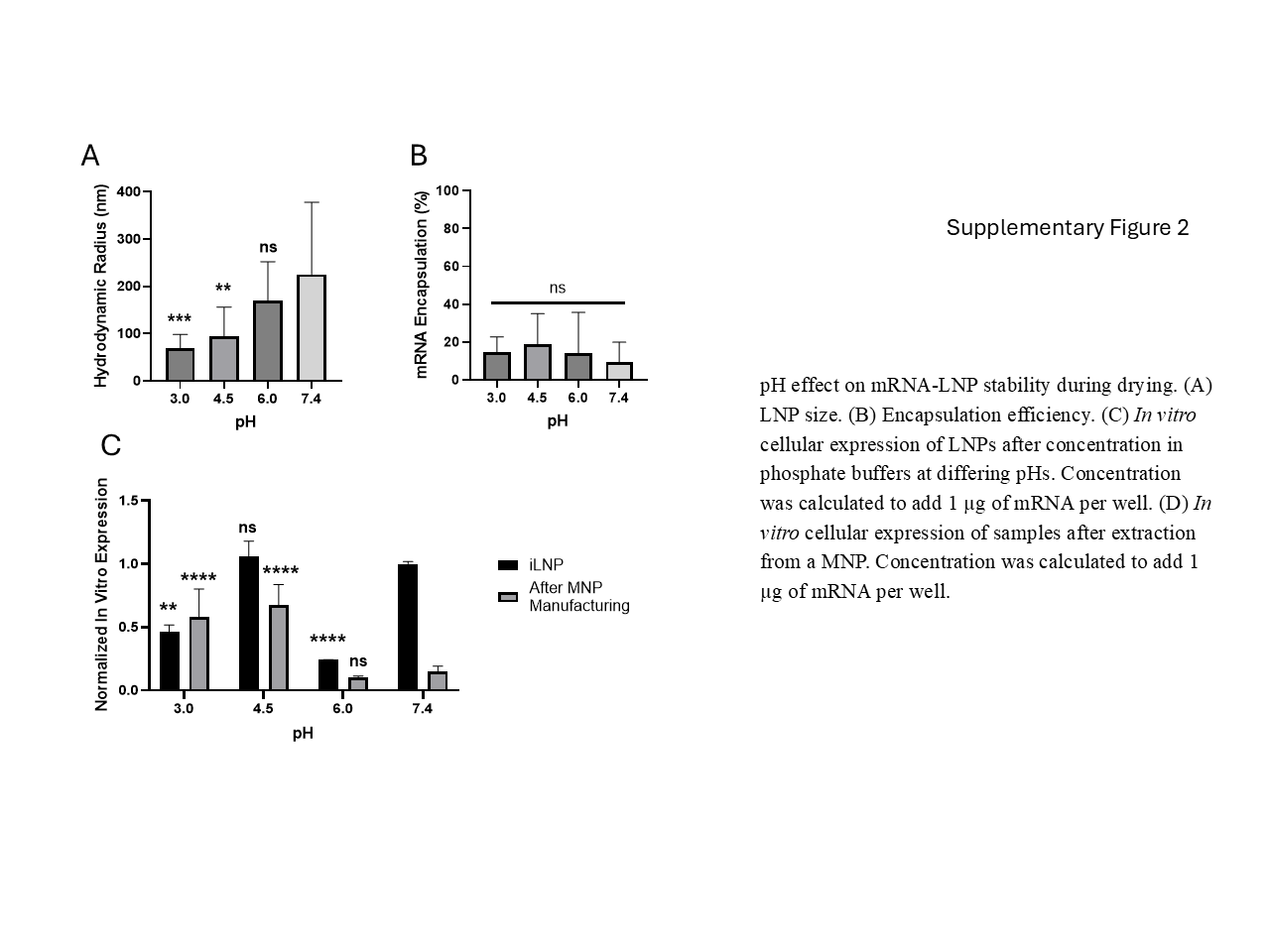


Supplementary Fig. 5: Effect of pH on mRNA-LNP stability after mRNA-LNP drying. (a) mRNA-LNP hydrodynamic radius. (b) mRNA encapsulation efficiency in LNPs. (c) *In vitro* cellular expression of luciferase-encoding mRNA-LNPs. mRNA-LNPs were concentrated in phosphate buffers at differing pHs, manufactured in MNPs, and reconstituted. Normalized to initial LNPs at pH 7.4. (n=4, ns = no significance, **p<0.01, ***p<0.001, ****p<0.0001, one-way ANOVA with multiple comparisons)


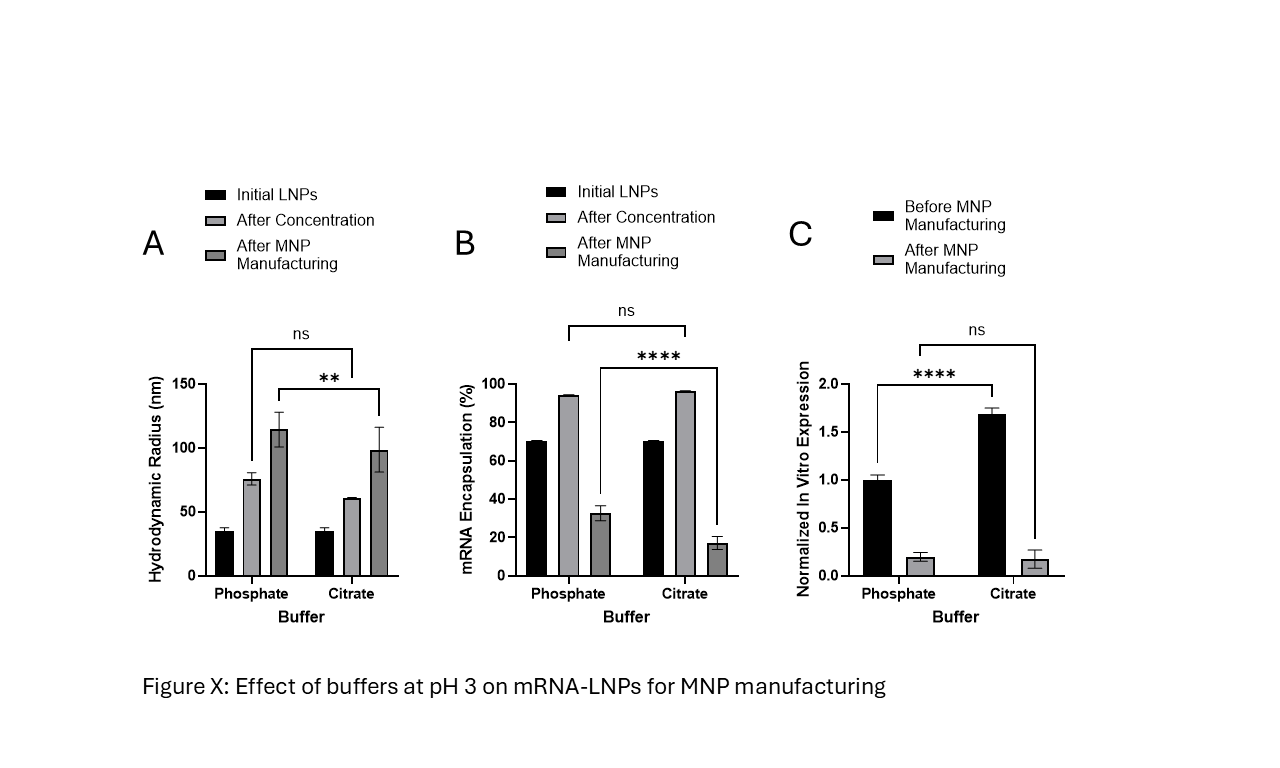


Supplementary Fig. 6: Effect of concentration buffer on mRNA-LNP stability in a MNP. (a) mRNA-LNP hydrodynamic radius. (b) mRNA encapsulation efficiency in LNPs. (c) *In vitro* cellular expression of luciferase-encoding mRNA-LNPs before and after reconstitution from a MNP. Normalized to initial LNPs in phosphate buffer. (n=2-3, ns=no significance, **p<0.01, ****p<0.0001, two-way ANOVA with multiple comparisons)


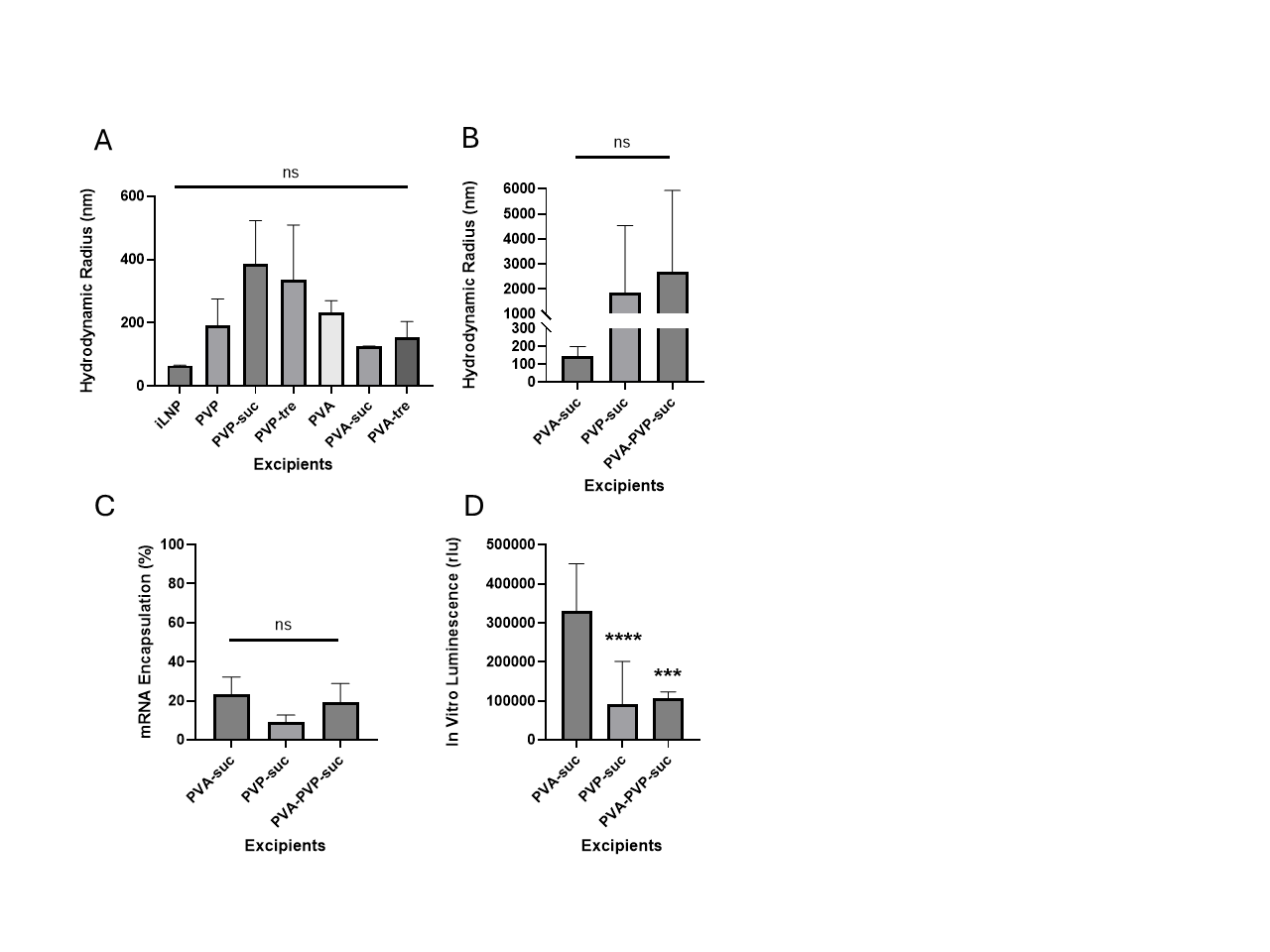


**Supplementary Fig. 7:** Effect of MNP formulation excipients. (a) polyA-LNP hydrodynamic radius and (b) mRNA-LNP hydrodynamic radius. (c) mRNA encapsulation efficiency in LNPs. (d) *In vitro* cellular expression of luciferase-encoding mRNA-LNPs. mRNA-LNPs were evaluated after reconstitution from MNPs formulated with polyvinyl alcohol (PVA) or polyvinylpyrrolidone (PVP) with or without sucrose (suc) or trehalose (tre). (n=2-8, ns = no significance, ***p<0.001, ****p<0.0001)


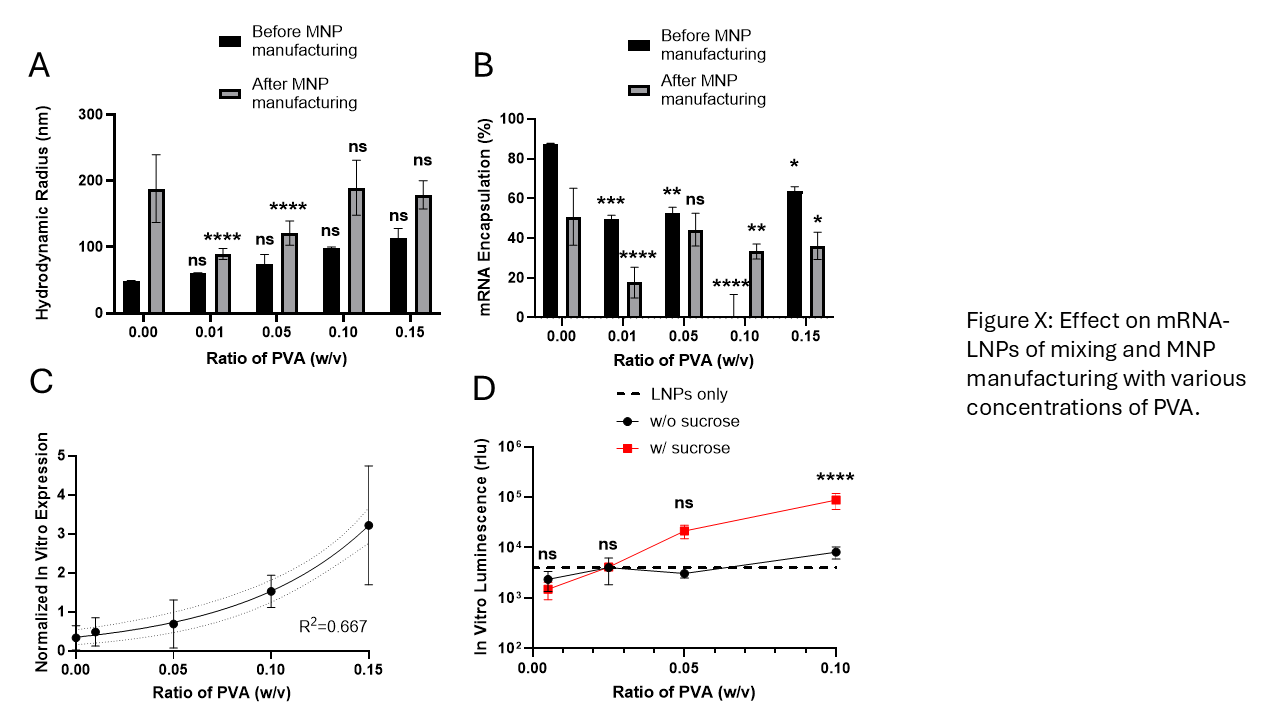


Supplementary Fig. 8: Effect of PVA content on mRNA-LNP MNP. (a) Hydrodynamic radius of mRNA-LNPs after reconstitution from MNPs containing 10% sucrose and 0%, 1%, 5%, 10%, or 15% PVA. (b) mRNA encapsulation in LNP after reconstitution from MNPs containing 10% sucrose and 0%, 1%, 5%, 10%, or 15% PVA. (c) In vitro expression of luciferase-encoding mRNA-LNPs after reconstitution from MNPs containing 10% sucrose and 0%, 1%, 5%, 10%, or 15% PVA in RAW 264.7 cells. (d) In vitro expression of luciferase-encoding mRNA-LNPs mixed with 1%, 2.5%, 5% or 10% PVA with or without 10% sucrose in RAW 264.7 cells. (n=3-6, ns=no significance, *p<0.05, **p<0.01, ***p<0.001, ****p<0.0001, two-way ANOVA, exponential fit with 95% confidence intervals)
